# A curative regimen would decrease HIV prevalence but not HIV incidence unless targeted to an ART-naïve population

**DOI:** 10.1038/srep22183

**Published:** 2016-02-24

**Authors:** Dobromir T. Dimitrov, Hans-Peter Kiem, Keith R. Jerome, Christine Johnston, Joshua T. Schiffer

**Affiliations:** 1 Vaccine and Infectious Disease Division, Fred Hutchinson Cancer Research Center, Seattle, Washington, USA; 2 Department of Applied Mathematics, University of Washington, Seattle, Washington, USA; 3 Clinical Research Division, Fred Hutchinson Cancer Research Center, Seattle, Washington, USA; 4 Department of Medicine, University of Washington, Seattle, Washington, USA; 5 Department of Laboratory Medicine, University of Washington, Seattle, Washington, USA

## Abstract

HIV curative strategies currently under development aim to eradicate latent provirus, or prevent viral replication, progression to AIDS, and transmission. The impact of implementing curative programs on HIV epidemics has not been considered. We developed a mathematical model of heterosexual HIV transmission to evaluate the independent and synergistic impact of ART, HIV prevention interventions and cure on HIV prevalence and incidence. The basic reproduction number was calculated to study the potential for the epidemic to be eliminated. We explored scenarios with and without the assumption that patients enrolled into HIV cure programs need to be on antiretroviral treatment (ART). In our simulations, curative regimes had limited impact on HIV incidence if only ART patients were eligible for cure. Cure implementation had a significant impact on HIV incidence if ART-untreated patients were enrolled directly into cure programs. Concurrent HIV prevention programs moderately decreased the percent of ART treated or cured patients needed to achieve elimination. We project that widespread implementation of HIV cure would decrease HIV prevalence under all scenarios but would only lower rate of new infections if ART-untreated patients were targeted. Current efforts to identify untreated HIV patients will gain even further relevance upon availability of an HIV cure.

HIV-1 replicates in activated CD4+ T-cells which results in gradual depletion of these cells over 8–10 years, leading to immunodeficiency and death^1^. Antiretroviral treatment (ART) suppresses viral replication and reverses disease progression^2^. While patients who are compliant with daily ART have relatively normal life expectancies^3^, non-replicating, latent HIV-1 persists in resting memory CD4+ T-cells by integrating into host chromosomal DNA^4^. Upon ART cessation, HIV-1 replication resumes within days to weeks, highlighting that ART is not curative and must be taken indefinitely^5^.

ART provides a public health benefit. In the absence of antiviral therapy, HIV-1 infected persons shed high levels of virus despite being largely asymptomatic, and are at high risk of transmitting HIV-1 to sexual partners. Suppression of replication dramatically decreases the risk of transmission^6^, and if implemented broadly even at late stages of disease, may lead to a substantial decrease in incidence over time^7^. Unfortunately, the effectiveness of this strategy can be limited in the context of medical non-compliance or resource constraints. Moreover, the cost of diagnosing and treating all infected persons is prohibitive in many countries.

There is recent increased optimism that an HIV-1 cure may be possible. The goal is to achieve complete eradication of latent provirus, or at least provide a functional cure, in which latent HIV-1 is lowered below a threshold necessary for reactivation, progression to AIDS, and transmission^8^. Most current strategies assume the need for elimination of replication with a short 3–6 month ART course prior to curative treatment. One idea is to use latency reversing agents (LRAs) that induce replication of latent HIV-1, which can then be expunged with ART^9^. Gene therapy may be employed to deliver DNA editing enzymes that mutate integrated HIV-1, thereby rendering these viruses replication incompetent^10^. Autologous transplantation of stem cells that are engineered *ex vivo* to be resistant to HIV-1 might allow a decrease in the size of the latent reservoir^11^, and also confer resistance to re-infection following functional cure.

Immune-based strategies are being considered as independent or complementary therapies^12^. Because ART causes HIV-1 T lymphocyte responses to wane over time^13^, immunologic approaches to cure may be more effective in the absence of ART. Therapeutic vaccines that boost the endogenous immunologic response, or transplantation of immune cells that are effective against HIV-1, may induce a functional or complete cure, and may partially protect against re-infection. Immunotoxins are antibodies engineered to have a lethal effect against HIV-1 infected cells and could theoretically be effective without suppressive ART^14^. Chimeric antigen receptor T-cells (CARs) are being engineered to target specific HIV epitopes that are expressed within latently and actively infected CD4+ T-cells^15^.

If one or more of these approaches is successful, then a comprehensive roll out strategy that accounts for scalability, cost and safety will be necessary. A critical consideration will be potential indirect benefits provided by curative approaches. A decrease in number of infected persons may lead to less incident infection. On the other hand, a widely available cure may repopulate the at risk susceptible pool, potentially leading to re-infections. Immunologic approaches to cure may limit this effect by protecting against re-infection. The addition of HIV prevention strategies which reduce the risk of HIV acquisition, such as vaccines or pre-exposure prophylaxis (PrEP), may also have synergistic effects on HIV incidence with curative interventions^16^. Finally, the nature of the implementation program may affect the epidemiologic impact of cure. Specifically, if cure is targeted towards patients already receiving comprehensive HIV care including ART then this may have different effects than cure programs that attempt to recruit and retain HIV positive patients who are not currently receiving ART.

Here we use mathematical models to forecast the effects of cure implementation programs on HIV-1 prevalence (proportion of individuals infected with HIV-1) and HIV-1 incidence (proportion of uninfected individuals who become infected annually). We identify that even slow introduction of cure approaches will decrease HIV-1 prevalence. However, only curative approaches that are implemented in ART-untreated patients will independently push epidemics towards extinction by limiting incident infections.

## Results

### Limited impact of cure on HIV-1 incidence if ongoing ART is a pre-requisite for cure

**Figure 1:**
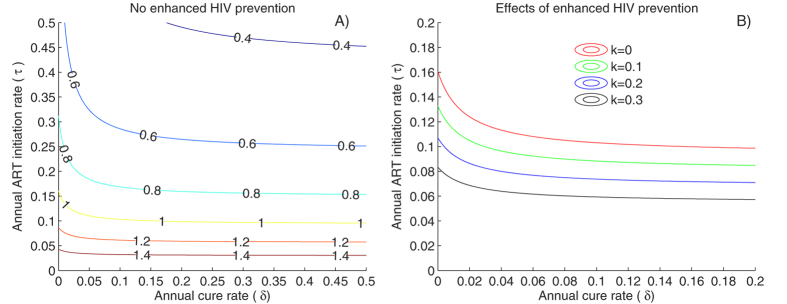
ART and HIV prevention coverage drive decreases in epidemic potential if only ART treated patients are eligible for cure interventions. (**A**) Values of the basic reproduction number (R_0_) assuming different annual treatment and cure rates assuming that enhanced HIV prevention is not available (k = 0). (**B**) Effects of HIV prevention coverage (k) on HIV eradication (curves represent R_0_ = 1). Under all scenarios, increasing annual cure rate has a negligible impact on R_0_.

We first assessed the impact of cure on HIV-1 epidemic trajectory with the assumption that cure regimens would only be available to patients already on ART. The logic underlying this assumption is that members of this patient population are readily accessible due to prior engagement in longitudinal care, and have fully-suppressed HIV viral load, which is likely to be prerequisite for eradication of the pool of HIV latently infected cells. Under this scenario, the percentage of patients receiving chronic ART had a far more powerful effect on lowering R_0_ than the percentage of ART-treated patients who also underwent curative treatment (Fig. 1A). Treatment alone resulted in R_0_ < 1 if 16% of untreated individuals initiated ART annually. However, because viral suppression on ART was the key step to breaking transmission chain, the addition of cure had little impact on R_0_ if fewer than 10% of HIV positive individuals initiated ART annually. Even if 50% of the ART-treated patients were cured annually, the ART initiation rate required for the elimination of HIV remained above 10%. In the context of increasing HIV prevention coverage such as PrEP or an HIV vaccine, fewer patients needed to receive ART to achieve R_0_ < 1 (Fig. 1B).

**Figure 2:**
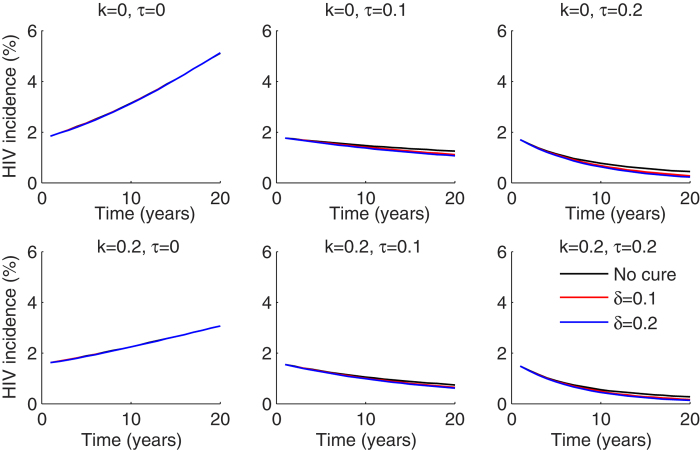
ART coverage is the most critical factor driving decreased HIV incidence if cure is available to only ART treated patients. Dynamics of HIV incidence measured as number of new HIV infections cases per 100 person years over 20 years for different combinations of HIV prevention coverage (k), ART initiation (τ) and cure (δ). Prevention programs are most effective at low treatment initiation rates per year.

Accordingly, HIV incidence over a 20 year period was virtually unaffected by the introduction of cure to ART treated patients (Fig. 2). An ART initiation rate of 10% of infected individuals per year resulted in a reduction in HIV incidence by 40% over 20 years, and 20% annual ART initiation rate nearly eliminated incident cases after 15–20 years. HIV prevention program implementation decreased incidence substantially, but only if ART introduction rates were low. High rates of HIV prevention did not augment the impact of concurrent cure interventions (Fig. 2).

**Figure 3:**
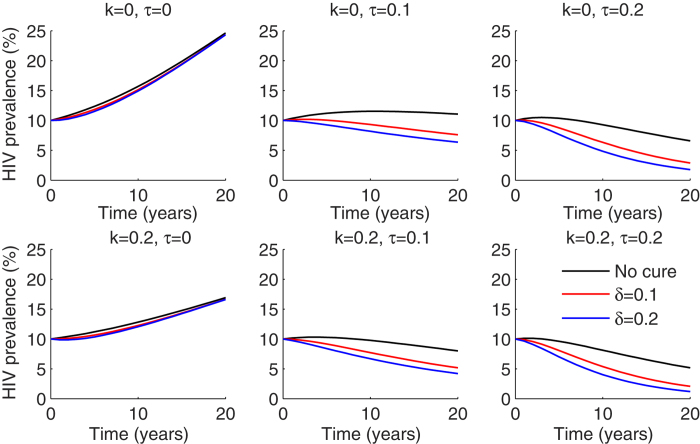
ART coverage and annual cure rate drive decreases in HIV prevalence if cure is available to only ART treated patients. Dynamics of HIV prevalence measured as percentage HIV infected in the population over 20 years for different combinations of HIV prevention coverage (k), ART initiation (τ) and cure (δ).

The addition of a curative regimen had a more significant impact on HIV prevalence, but only if the percentage of ART-treated patients was high (Fig. 3). Assuming an ART initiation rate of 10% per year, 2–5% absolute decreases in prevalence due to HIV cure, were noted 5 years after introduction into the population. HIV prevention programs were capable of slowing the increase in HIV prevalence, though these effects were less pronounced in the context of widespread ART implementation. Twenty percent coverage with an HIV prevention strategy resulted in an almost 30% decrease in prevalence over 20 years without addition of cure or ART (Fig. 3). These results suggest that curative regimens, which are predicated on viral load suppression with ART, will not limit new cases. However, they will limit the number of patients needing lifelong ART if implemented in the context of high ART use. Prevention programs will have highest utility in low ART and cure scenarios.

### Significant impact of cure on HIV-1 incidence if ongoing ART is not a pre-requisite for enrollment in cure programs

**Figure 4:**
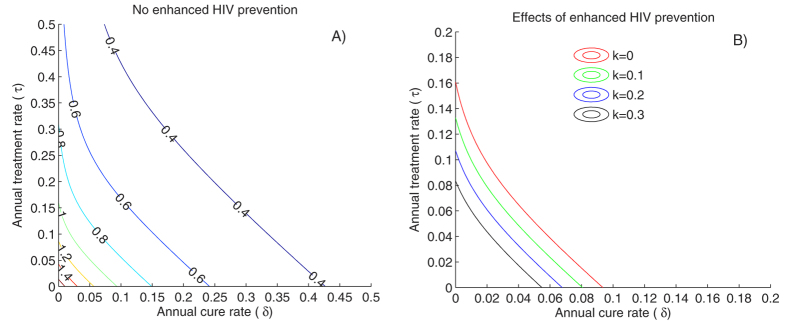
ART coverage, annual cure rate and HIV prevention coverage drive decreases in epidemic potential if ART untreated patients are recruited directly into cure programs. (**A**) Values of the basic reproduction number (R_0_) assuming different annual treatment and cure rates assuming that enhanced HIV prevention is not available (k = 0). (**B**) Effects of HIV prevention coverage (k) on HIV eradication (curves represent R_0_ = 1).

We next assessed the impact of HIV cure regimens on a local HIV epidemic trajectory with the assumption that cure would be offered to all HIV-positive individuals including those not yet receiving suppressive ART. Under this scenario, both the percentage of patients offered ART, and the percentage of patients offered curative treatment lowered R_0_ (Fig. 4A). Annual ART and cure rates of 6% would be sufficient to reduce R_0_ < 1 with curative treatment rates of 10% alone being sufficient to eliminate the local HIV epidemic. In the context of increasing HIV prevention efforts, fewer patients would be required to receive ART or curative treatment to achieve R_0_ < 1 (Fig. 4B).

**Figure 5:**
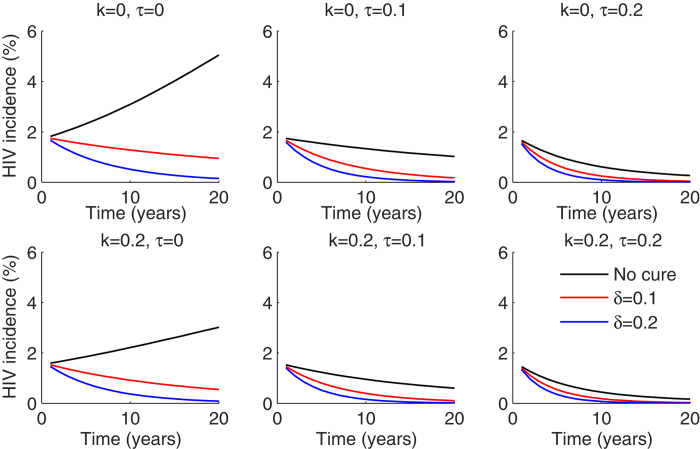
ART and cure coverage drive decreased HIV incidence if ART untreated patients are recruited directly into cure programs. Dynamics of HIV incidence measured as number of new HIV infections cases per 100 person years over 20 years for different combinations of HIV prevention coverage (k), ART initiation (τ) and cure (δ).

The addition of curative therapy also had a dramatic impact on incidence irrespective of ART or HIV prevention rates. Even without enhanced HIV prevention or ART, the opportunity to cure 10% of the infected individuals annually had the potential to reduce HIV incidence by 50% over 20 years. Combining 10% ART and curative rates per year resulted in more than an 80% decrease in incidence compared to treatment alone. In absolute terms the impact of the curative treatment was stronger when few or no infected persons were on ART (Fig. 5). We also ran simulations assuming 3- or 6-month delays between implementation of a cure program based on need for a short course of suppressive ART (Supplementary Fig. S2). This did not significantly alter our results, other than to slightly delay the decrease in HIV prevalence (Supplementary Fig. S3).

**Figure 6:**
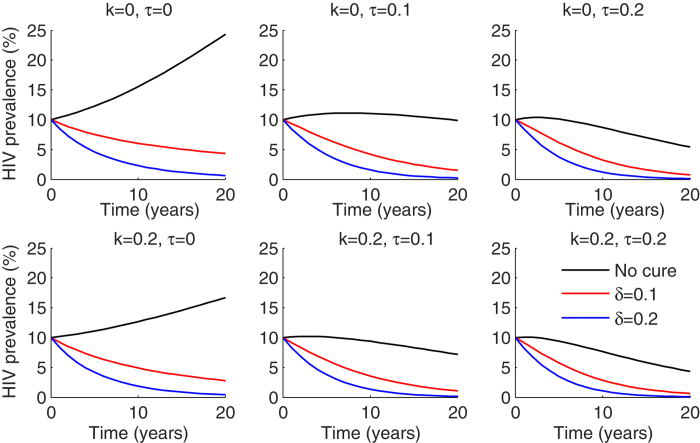
ART and cure coverage drive decreased HIV prevalence if ART untreated patients are recruited directly into cure programs. Dynamics of HIV prevalence measured as percentage HIV infected in the population over 20 years for different combinations of HIV prevention coverage (k), ART initiation (τ) and cure (δ).

Similarly, the addition of a curative regimen had a substantial impact on HIV prevalence, regardless of the percentage of patients on ART (Fig. 6). Overall, these results suggest that curative regimens, which target HIV infected patients who are not currently on ART, would have an important impact on both HIV incidence and prevalence over relatively short time frames, even if implemented in populations with low use of ART and HIV prevention.

### Impact of curative regimens that partially protect against re-infection

An immunotherapeutic regimen might also have the beneficial effect of decreasing probability of HIV acquisition per sex act, which we investigated with a modified model (Supplementary Fig. S4). Regardless of whether ART treated or untreated patients were targeted for these regimens, a partial protection (up to 60%) of the cured individuals against reinfection with HIV had only a small effect on projected HIV prevalence and HIV incidence over 20 years (Supplementary Figs S5 and S6) even if the protection lasted 10 years. The impact of the potential reduction in susceptibility of cured individuals is negligible in comparison with the reduction in infectiousness provided by curative regimens.

### Sensitivity Analysis

We tested scenarios in which ART did not uniformly achieve HIV suppression. When ART was only 90% effective in the absence of cure, HIV eradication was impossible even if 35% of untreated initiated ART annually (Supplementary Fig. S7). HIV incidence was unchanged by cure under this assumption while HIV prevalence continued to decrease as a function of increased cure (Supplementary Figs S8 and S9). Scenarios with lower treatment rates (3–6% annually) demonstrated no impact on HIV incidence and less pronounced reductions in HIV prevalence (see Supplementary Figs S10–S12).

## Discussion

While an HIV-1 cure is currently lacking, there is optimism that one or some combination of developing strategies will allow eradication or long-term functional cure. If a scalable curative approach emerges, then a critical question is how roll out will impact the epidemic. We use mathematical models to demonstrate that curative approaches will only significantly impact the basic reproductive number and HIV-1 incidence, if programs are targeted towards HIV patients who are not yet on ART. Cure programs targeted towards ART treated patients only will have a substantial impact on prevalence if ART use is widespread, but will decrease incidence to a minor extent beyond ART alone. This highlights the potent prevention impact already provided by ART. For HIV-infected patients not yet on ART, a several month course of ART will likely be necessary prior to curative treatments. For these reasons, research efforts geared towards HIV-1 cure should develop in parallel to and not at the expense of ART provision. Moreover, if cure implementation programs are developed, it will be crucial to target HIV-infected but undiagnosed and/or untreated patients.

Our results reinforce the prediction that broad utilization of ART in the HIV-infected community will powerfully lower HIV incidence. In our model, HIV-1 prevention efforts such as vaccination, or PrEP are predicted to also be important for limiting HIV incidence and prevalence. The results of this analysis are similar to projections from other modeling studies which evaluate combined effects of expanded ART and HIV prevention^17^,^18^,^19^ and show that the relative impact of prevention programs will decrease with more widespread use of ART and cure of ART untreated, HIV infected persons. We conclude that the degree of funding provided for prevention programs in at risk populations, should depend in part on extent of coverage provided by ART and HIV cure programs. If these medical services are provided to a large proportion of HIV infected persons, then prevention programs will provide less added benefit.

As curative strategies are gradually introduced, HIV primary care clinics will continue to play a critical role. Implementation of cure will build upon existing clinical and public health infrastructure for identifying untreated HIV patients. Because of difficulty measuring the HIV latent reservoir in tissue sanctuaries of infection, functionally cured patients will need to be followed indefinitely to ensure no HIV reactivation^9^,^20^. HIV prevention strategies will need to be included as the standard package of care to prevent HIV re-infection. Despite continued need for routine follow up, cure programs will provide cost savings, as patients will no longer need to remain on suppressive therapy for life.

Our model suggests that an immunologic HIV-1 cure that prevents re-infection, while beneficial for an individual patient will have little impact on incidence. This implies that most new HIV infections would occur in previously uninfected persons. This finding should not dissuade efforts to develop immunotherapies for HIV that are both therapeutic and preventative, but does reinforce the fact that HIV prevention efforts need to be targeted to the entire at risk population, not just recently cured patients.

We intended to identify general trends that would emerge following introduction of a cure program. The model is not designed to make precise quantitative predictions about specific epidemics. The impact of HIV-1 cure programs will differ according to epidemic context. For instance, in many communities, the most significant spreaders of HIV-1 are also the most difficult to consistently engage in care. This may preclude elimination of HIV-1 despite comprehensive and integrated local ART and cure programs.

We predict that implementation of curative HIV-1 therapies will have a beneficial impact on HIV-1 incidence only if patients who are currently not receiving ART are targeted. Development of HIV-1 cure technologies should evolve in parallel with ongoing research to identify, engage and treat HIV-1 infected persons who are unaware of their diagnosis, or have yet to seek treatment.

## Methods

### Transmission model

We developed a compartmental mathematical model of HIV transmission in a sexually active population and used it to evaluate the impact of ART, HIV prevention programs, and cure on the HIV epidemic in Sub-Saharan Africa (see Supplementary Fig. S1). The population is stratified in compartments by HIV status (HIV-negative, HIV-positive, and AIDS). The HIV-negative individuals are divided into groups with respect to their exposure to HIV prevention programs, while HIV-positive persons are stratified according to ART. HIV-negative adolescents who become sexually active are assumed to join the community at a constant rate, which is selected to ensure 1% population growth representative for South Africa. The rates at which individuals acquire HIV, i.e., forces of infections for different classes are derived from standard binomial models based on number of partners per susceptible person, number of sex acts per partnership, HIV acquisition risk per sex act and efficacy of HIV prevention and ART in reducing the susceptibility and infectiousness. A complete description of the model and its mechanisms is provided in the Supplementary Information.

### Epidemic settings

We use epidemiological data representative of regions with high HIV prevalence in sub-Saharan Africa^21^. Acquisition probabilities per vaginal act with HIV infected partner are obtained from meta-analyses of the observational data from these countries^22^. Demographic and sexual behavior characteristics including average number of partners per year, frequency of sex acts, fraction of infected, and lifetime duration of sexual activity are estimated from published data for South Africa^23^,^24^. A full description of the parameter selection process is given in the Supplementary Information.

### Coverage and effectiveness of HIV prevention, ART and curative treatment

In our simulations, twenty percent of the initially infected population is assumed to be receiving ART^21^. Treated individuals who strictly follow prescribed daily regimens are assumed to have a 95% reduction in infectiveness relative to non-treated individuals^6^. Accounting for possible treatment interruptions among ART recipients, we consider scenarios assuming a lower (90%) efficacy of ART in reducing the risk of HIV transmission per sexual act^25^. A varying fraction of the initial and newly recruited population is assumed to be covered by an HIV prevention program such as vaccination or PrEP, and to strictly follow the prescribed regimens. Clinical trial results suggest that oral PrEP provides 60–70% protection against HIV acquisition in Africa when adherence is high^26^,^27^. An HIV-1 vaccine demonstrated 31% efficacy in the RV144 trial^28^ but 50% reduction in acquisition over an 18–36 month time period will be required for regulatory approval^28^,^29^. Here we assume that the susceptibility of the individuals using HIV prevention is reduced by 60%.

Since curative treatment is not currently available we make speculative assumptions about the characteristics of such an intervention. We explore a scenario in which candidates for HIV cure are recruited exclusively from ART treatment programs, and a scenario in which HIV infected persons who are not receiving ART are targeted for cure. If ART untreated patients are eligible for cure, then we also assume that ART pre-treatment for 3 or 6 months will be necessary to fully eliminate circulating virus prior to cure implementation. Unless otherwise stated, we assume that cured individuals return to the susceptible population and can be re-infected with HIV. Scenarios assuming partial protection of the cured individuals against reinfection are also considered.

### Evaluation metrics

The basic reproduction number (R_0_) is defined as the number of secondary infections a typical single infected individual will cause in a population with no immunity to the disease in the absence of interventions to control the infection. R_0_ determines whether or not an infectious disease will spread through a population. In general, if R_0_ > 1 the infection persists in the population while if R_0_ < 1 then the infection may be eradicated^30^. We calculate R_0_ for different combinations of HIV prevention, ART and cure to evaluate their potential to eliminate the HIV epidemic. Because decreases in R_0_ may manifest over short or long time intervals, we also analyze the impact of the addition of curative treatment on the HIV prevalence and incidence. A short delay (3–6 months) in cure implementation due to need for antecedent ART suppression is included in our analyses of HIV incidence and prevalence, but does impact R_0_, and is not included in these calculations.

## Additional Information

**How to cite this article**: Dimitrov, D. T. *et al.* A curative regimen would decrease HIV prevalence but not HIV incidence unless targeted to an ART-naïve population. *Sci. Rep.* **6**, 22183; doi: 10.1038/srep22183 (2016).

## Supplementary Material

Supplementary Information
