# Supplementary material for: A curative regimen would decrease HIV prevalence but not HIV incidence unless targeted to an ART-naïve population
Source: Sci Rep. 2016 Feb 24;6:22183. doi: 10.1038/srep22183 (PMC4764923; doi:10.1038/srep22183)
Supplement: Supplementary Information [file srep22183-s1.doc]

**Supplementary information**

**A curative regimen would decrease HIV prevalence but not HIV incidence unless targeted to an ART-naïve population**

Dobromir T. Dimitrov^1,2,*^, Hans-Peter Kiem^3,4^, Keith R. Jerome^1,5^, Christine Johnston^4^, Joshua T. Schiffer^1,4^

^1^Vaccine and Infectious Disease Division, Fred Hutchinson Cancer Research Center, Seattle, Washington, USA

^2^Department of Applied Mathematics, University of Washington, Seattle, Washington, USA

^3^Clinical Research Division, Fred Hutchinson Cancer Research Center, Seattle, Washington, USA

^4^Department of Medicine, University of Washington, Seattle, Washington, USA

^5^Department of Laboratory Medicine, University of Washington, Seattle, Washington, USA

^*^Corresponding author: [ddimitro@fredhutch.org](mailto:ddimitro@fredhutch.org)

**Models description, parameterization, additional results and sensitivity analyses.**

**Description of the main Model**


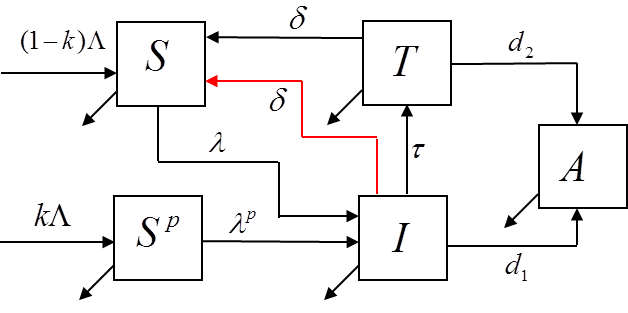


**Figure S1 Flow diagram of the model of HIV transmission assuming that cured individuals are fully susceptible to reinfection with HIV**. Simulated population is stratified in compartments by HIV, prevention use and treatment status as susceptibles not using prevention (S), susceptibles using prevention (S^p^), infected not on ART (I), infected on ART (T),and individuals who succumb to AIDS (A). Scenarios in which ART is or not required for cure are compared by allowing or not the untreated infected (I) to be cured (red arrow).

*S*- susceptible not covered by HIV prevention (vaccine or PrEP)

*S^p^*- susceptible covered by HIV prevention (vaccine or PrEP)

*I* - HIV-positive untreated

*T* - HIV-positive on ART

*A*- individuals who developed AIDS

*Λ*- adolescents reaching sexual maturity

*k*- HIV prevention coverage

*τ*- rate to initiate ART among HIV-positive individuals

*δ*- fraction of the HIV-positive individuals cured annually

*d_1_, d_2_*- rates to develop AIDS if untreated or on ART

- average time to remain sexually active

$$\begin{matrix} \frac{dS}{dt}=(1-k)\Lambda+\delta T+\delta I-(\lambda+\mu)S \\ \frac{dS^{p}}{dt}=k\Lambda-(\lambda^{p}+\mu)S^{p} \\ \frac{dI}{dt}=\lambda S+\lambda^{p}S^{p}-\delta I-(\mu+d_{1}+\tau)I \\ \frac{dT}{dt}=\tau I-\delta T-(\mu+d_{2}) T \\ \frac{dA}{dt}=d_{1}I+d_{2}T \end{matrix}$$

- forces of infection for individuals who use PrEP or not. Calculated as:

**# partners per year** *x* **acquisition risk per partnership with HIV-positive individual from a specific class** *x* **prevalence of the class in the overall populations**

$$\begin{matrix} \lambda=\rho\left((1-(1-\beta)^{n/\rho})\frac{I}{N}+(1-(1-(1-\alpha_{T})\beta)^{n/\rho})\frac{T}{N}\right) \\ \lambda^{p}=\rho\left((1-(1-(1-\alpha_{p})\beta)^{n/\rho})\frac{I}{N}+(1-(1-(1-\alpha_{p})(1-\alpha_{T})\beta)^{n/\rho})\frac{T}{N}\right) \end{matrix}$$

- average number of sex partners per year

*β*- HIV acquisition risk per sex act with untreated HIV positive partner

*n*- number of sex acts per year

*α_p_*- Efficacy of HIV prevention in reducing HIV susceptibility per act

*α_T_* - ART efficacy in reducing HIV infectiousness per act

*N*- sexually active individuals (all classes except compartment A)

**Model parameterization**

**Table S1.** Parameter values and ranges used in the main analysis

| **Parameter** | **Description** | **Values and ranges** | **Ref.** |
| --- | --- | --- | --- |
| 1. **Behavioral and epidemic parameters** | |  |  |
| β | HIV acquisition risk per sexual act | 0.003 |  |
| μ | Annual departure rate based on background mortality and average time to remain sexually active | 0.0333 |  |
| d_r_ (d_2_) | HIV-related mortality rates for untreated (treated) individuals | 10% (5%) |  |
| n | Average number of sexual acts per year | 80 |  |
| ρ | Average number of sexual partners per | 1.2 |  |
|  | Initial HIV prevalence | 10% | assumed |
|  | Initial fraction of infected on ART | 20% |  |
| 1. **Intervention parameters** | | **Baseline value** |  |
| k | HIV prevention coverage. Proportion of susceptibles vaccinated or using PrEP. | 0-20% | assumed |
| α_p_ | HIV prevention efficacy in reducing HIV susceptibility per act | 60% |  |
| α_T_ | ART efficacy in reducing HIV infectiousness per act | 95% |  |
| *τ* | Rate to initate ART among HIV-positive individuals | 0-20% | assumed |
| *δ* | Proportion of the HIV-positive (only treated in some scenarios) individuals cured annually | 0-20% | assumed |

**Model modification assuming that infected individuals need a short course (3-6 months) of antecedent ART suppression to be cured**


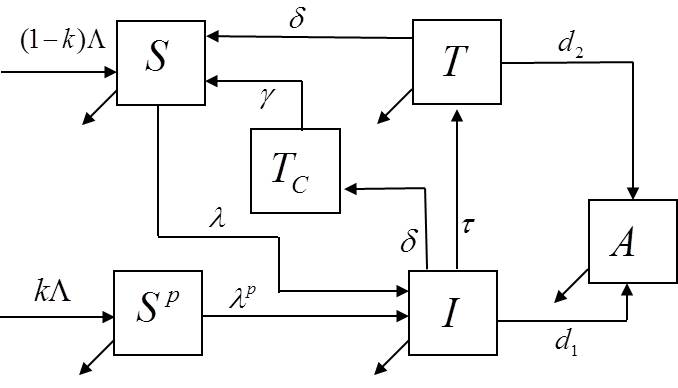


**Figure S2. Flow diagram of the model of HIV transmission assuming that infected individuals need a short course of ART**. Simulated population is stratified in compartments by HIV, prevention use and treatment status as susceptibles not using prevention (S), susceptibles using prevention (S^p^), infected not on ART (I), infected on ART (T), individuals enrolled in the curative program (T_C_) and individuals who succumb to AIDS (A). We assume that only short course of ART is required for cure.

*S*- susceptible not covered by HIV prevention (vaccine or PrEP)

*S^p^*- susceptible covered by HIV prevention (vaccine or PrEP)

*I* - HIV-positive untreated

*T* - HIV-positive on ART

*T_C_* – individuals on short course ART as a part of curative regimen

*A*- individuals who developed AIDS

*Λ*- adolescents reaching sexual maturity

*k*- HIV prevention coverage

*τ*- rate to initiate ART among HIV-positive individuals

*δ*- fraction of infected individuals cured annually

γ- untreated individuals cured after completing a short course ART as a part of curative regimen

*d_1_, d_2_*- rates to disease progression to AIDS if untreated or on ART

- average time to remain sexually active

$$\begin{matrix} \frac{dS}{dt}=(1-k)\Lambda+\delta T+\gamma T_{C}-(\lambda+\mu)S \\ \frac{dS^{p}}{dt}=k\Lambda-(\lambda^{p}+\mu)S^{p} \\ \frac{dI}{dt}=\lambda S+\lambda^{p}S^{p}-(\delta+\mu+d_{1}+\tau)I \\ \frac{dT}{dt}=\tau I-(\delta+\mu+d_{2}) T \\ \frac{dT_{C}}{dt}=\delta I-(\mu+d_{2}+\gamma) T_{C} \\ \frac{dA}{dt}=d_{1}I+d_{2}(T+T_{C}) \end{matrix}$$

Forces of infection for individuals who use HIV prevention (λ^p^) or neither (λ) are calculated as follows:

$$\begin{matrix} \lambda=\rho\left((1-(1-\beta)^{n/\rho})\frac{I}{N}+(1-(1-(1-\alpha_{T})\beta)^{n/\rho})\frac{T+T_{C}}{N}\right) \\ \lambda^{p}=\rho\left((1-(1-(1-\alpha_{p})\beta)^{n/\rho})\frac{I}{N}+(1-(1-(1-\alpha_{p})(1-\alpha_{T})\beta)^{n/\rho})\frac{T+T_{C}}{N}\right) \end{matrix}$$

- average number of sex partners per year

β - HIV acquisition risk per sex act with untreated HIV positive partner

*n*- number of sex acts per year

*α_p_, α_c_*- efficacy of PrEP and cure in reducing HIV susceptibility per act

α_T_ - ART efficacy in reducing HIV infectiousness per act

*N*- sexually active individuals (all classes except A)


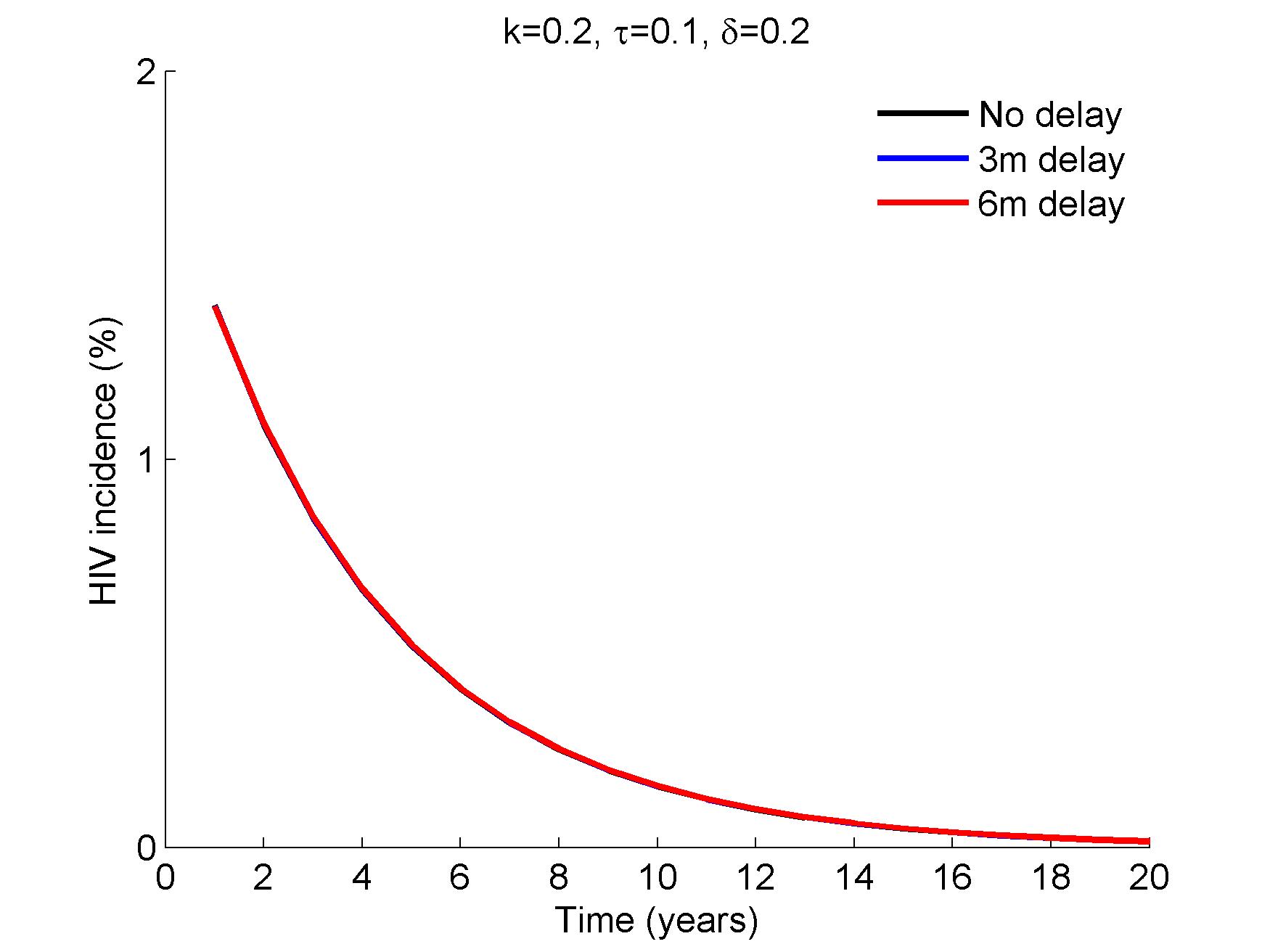

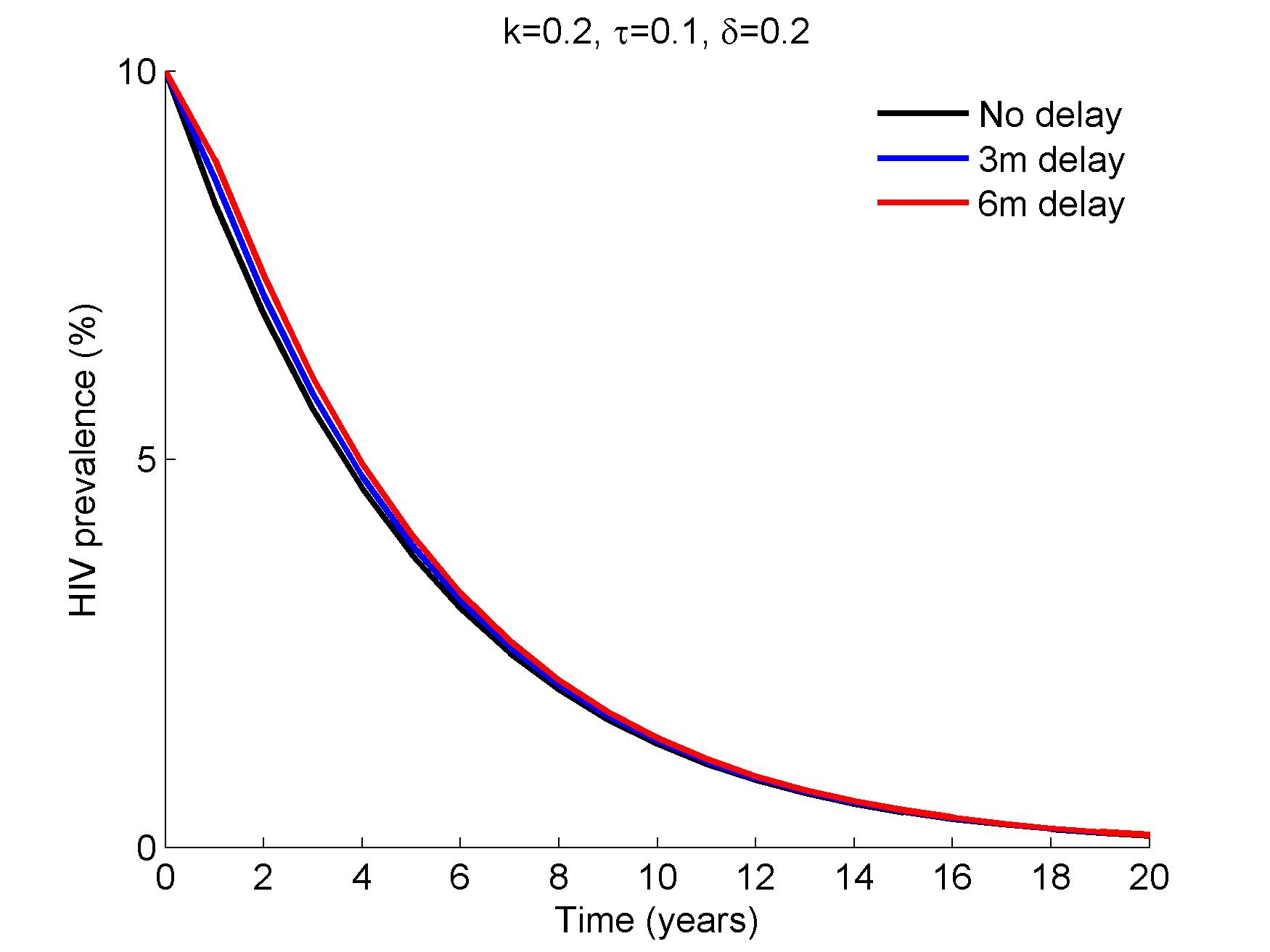


A)

B)

**Figure S3. Scenarios assuming that infected individuals need a short course of ART to be cured.** Dynamics of A) HIV incidence and B) HIV prevalence over 20 years assuming that 20% of all HIV infected individuals initiate curative regimens annually, 20% of the uninfected use enhanced HIV prevention (k=0.2) and 10% of the untreated individuals initiate ART annually (τ=0.1). **Insignificant delay in the HIV prevalence reduction is observed but HIV incidence is not impacted.**

**Model modification assuming that cured individuals are partially and temporally protected from reinfection with HIV**


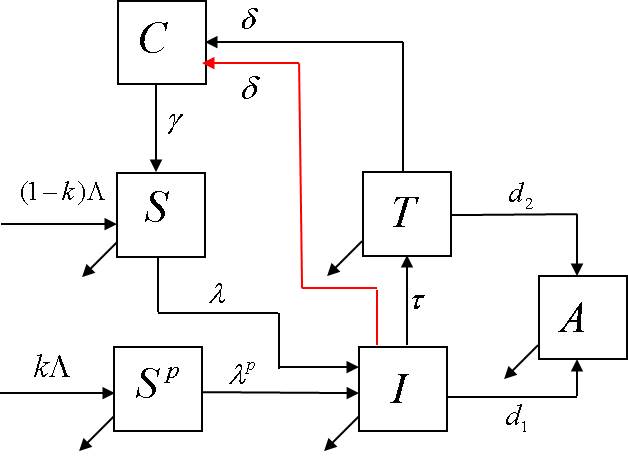


**Figure S4. Flow diagram of the model of HIV transmission assuming that cured individuals are partially protected against reinfection with HIV**. Simulated population is stratified in compartments by HIV, PrEP use and treatment status as susceptibles not using PrEP (S), susceptibles using PrEP (S^p^), infected not on treatment (I), infected on ART (T), cured individuals (C) and individuals who succumb to AIDS (A). Scenarios in which ART is or not required for cure are compared by allowing or not the untreated infected (I) to be cured (red arrow).

*S*- susceptible not covered by HIV prevention (vaccine or PrEP)

*S^p^*- susceptible covered by HIV prevention (vaccine or PrEP)

*I* - HIV-positive untreated

*T* - HIV-positive on ART

*C* – individual cured from HIV

*A*- individuals who developed AIDS

*Λ*- adolescents reaching sexual maturity

*k*- HIV prevention coverage

*τ*- rate to initiate ART among HIV-positive individuals

*δ*- fraction of treated (and untreated in some scenarios) individuals cured annually

γ- fraction of the cured individuals losing protection against reinfection with HIV annually

*d_1_, d_2_*- rates to disease progression to AIDS if untreated or on ART

- average time to remain sexually active

$$\begin{matrix} \frac{dS}{dt}=(1-k)\Lambda+\gamma C-(\lambda+\mu)S \\ \frac{dS^{p}}{dt}=k\Lambda-(\lambda^{p}+\mu)S^{p} \\ \frac{dI}{dt}=\lambda S+\lambda^{p}S^{p}+\lambda^{c}C-\delta I-(\mu+d_{1}+\tau)I \\ \frac{dT}{dt}=\tau I-\delta T-(\mu+d_{2}) T \\ \frac{dC}{dt}=\delta T+\delta I-(\lambda^{c}+\mu+\gamma) C \\ \frac{dA}{dt}=d_{1}I+d_{2}T \end{matrix}$$

Forces of infection for individuals who use PrEP (λ^p^), cured (λ^c^) or neither (λ). Calculated as:

**# partners per year** *x* **acquisition risk per partnership with HIV-positive individual from a specific class** *x* **prevalence of the class in the overall populations**

$$\begin{matrix} \lambda=\rho\left((1-(1-\beta)^{n/\rho})\frac{I}{N}+(1-(1-(1-\alpha_{T})\beta)^{n/\rho})\frac{T}{N}\right) \\ \lambda^{p}=\rho\left((1-(1-(1-\alpha_{p})\beta)^{n/\rho})\frac{I}{N}+(1-(1-(1-\alpha_{p})(1-\alpha_{T})\beta)^{n/\rho})\frac{T}{N}\right) \\ \lambda^{c}=\rho\left((1-(1-(1-\alpha_{c})\beta)^{n/\rho})\frac{I}{N}+(1-(1-(1-\alpha_{c})(1-\alpha_{T})\beta)^{n/\rho})\frac{T}{N}\right) \end{matrix}$$

- average number of sex partners per year

β - HIV acquisition risk per sex act with untreated HIV positive partner

*n*- number of sex acts per year

*α_p_, α_c_*- efficacy of PrEP and cure in reducing HIV susceptibility per act

α_T_ - ART efficacy in reducing HIV infectiousness per act

*N*- sexually active individuals (all classes except A)


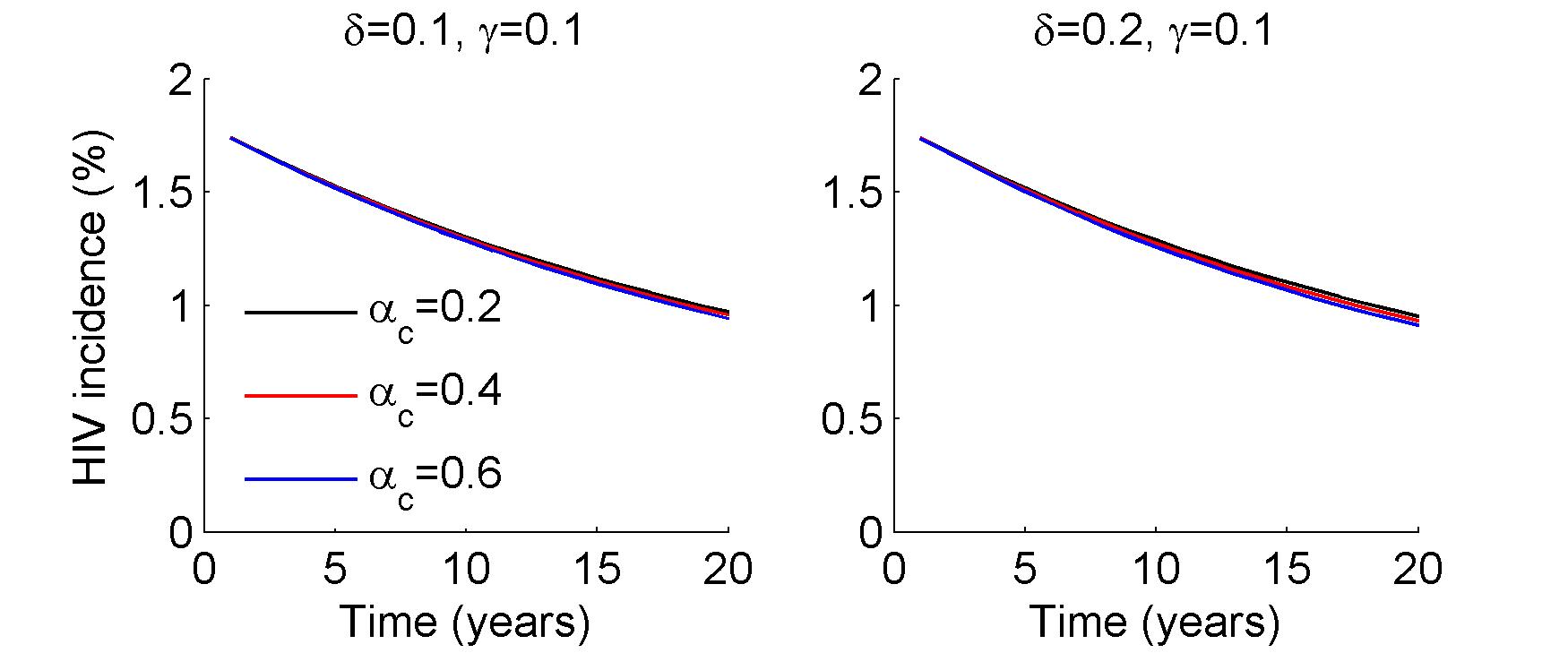


A)

B)

**Cure is contingent upon ART**

**Cure is not contingent upon ART**


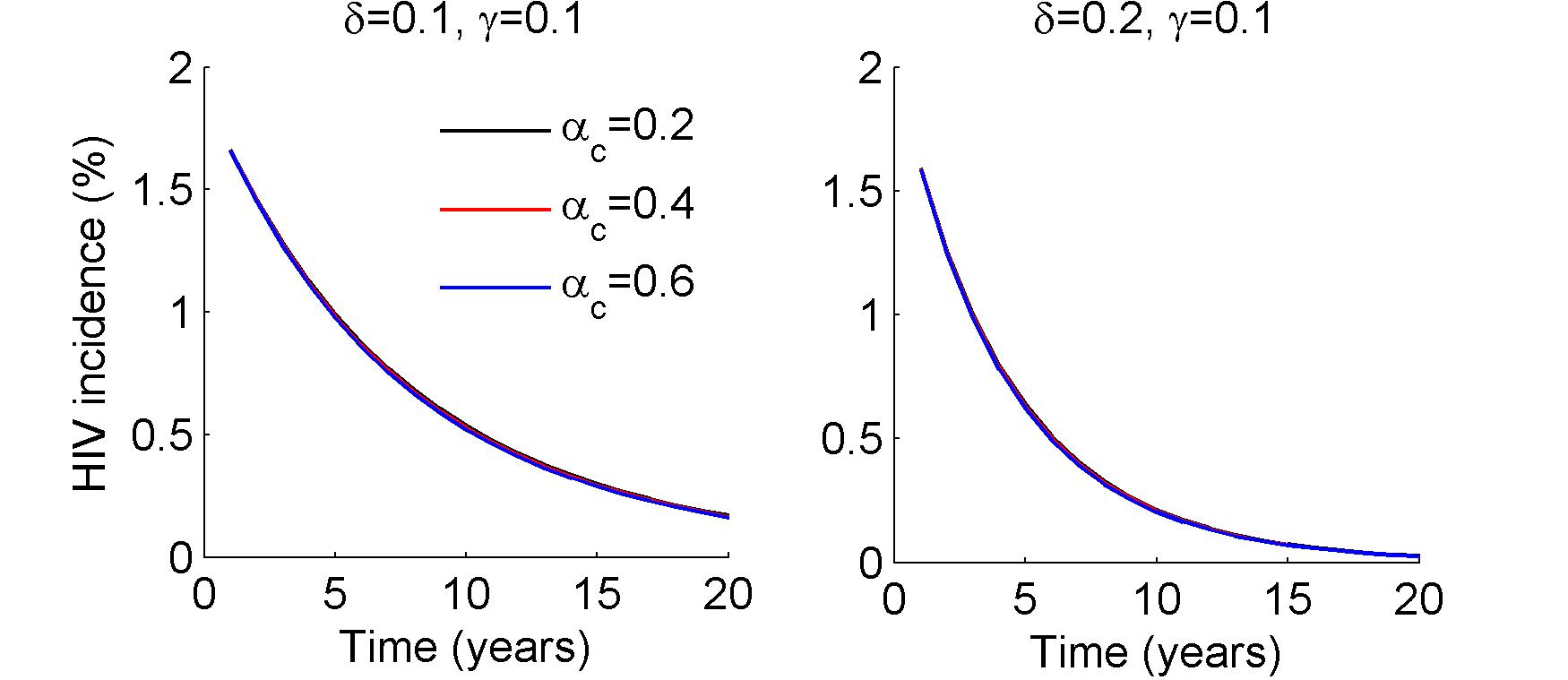


C)

D)

**Figure S5. Scenarios assuming that cured individuals are partially protected against reinfection with HIV.** Dynamics of HIV incidence over 20 years when A,C) 10% and B,D) 20% of the patients on ART (top) or of all HIV infected (bottom) are cured annually and cured individuals are partially protected against reinfection for 10 years. It is also assumed that enhanced HIV prevention is not available (k=0) and that 10% of the untreated individuals initiate ART annually (τ=0.1).


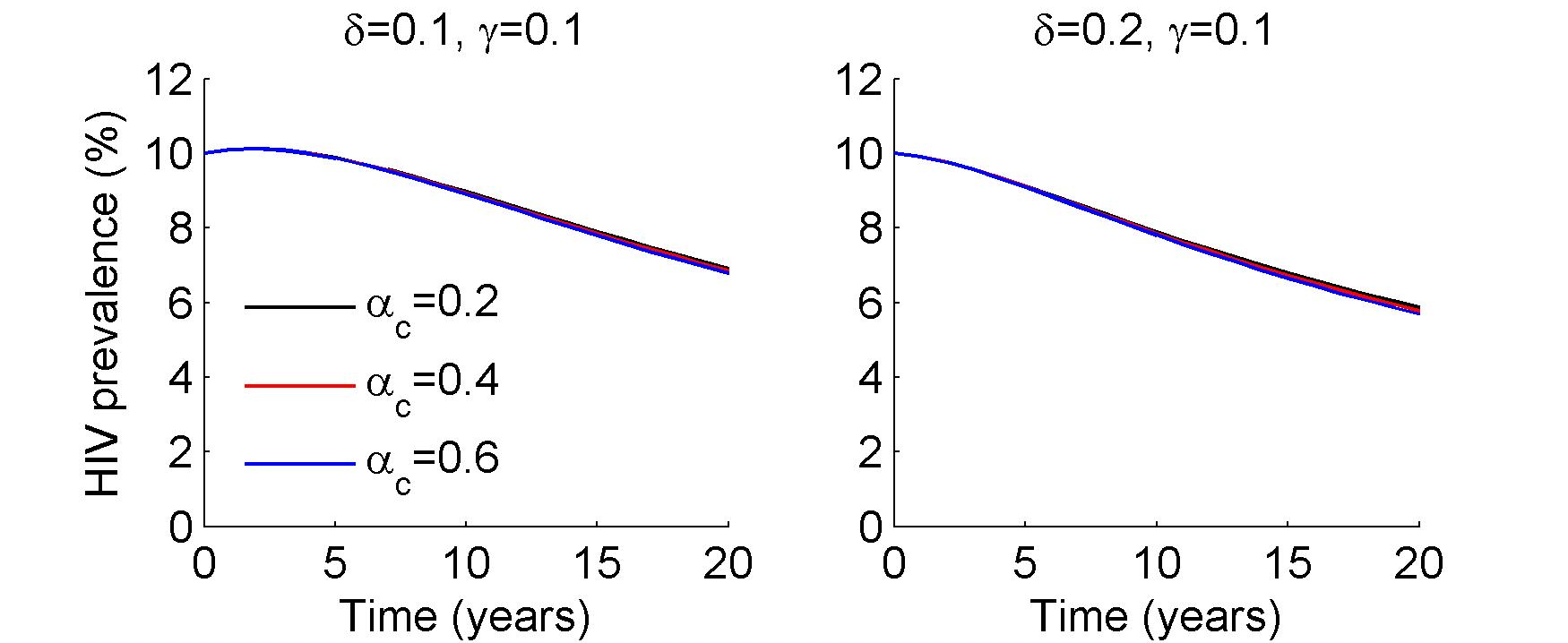


A)

B)


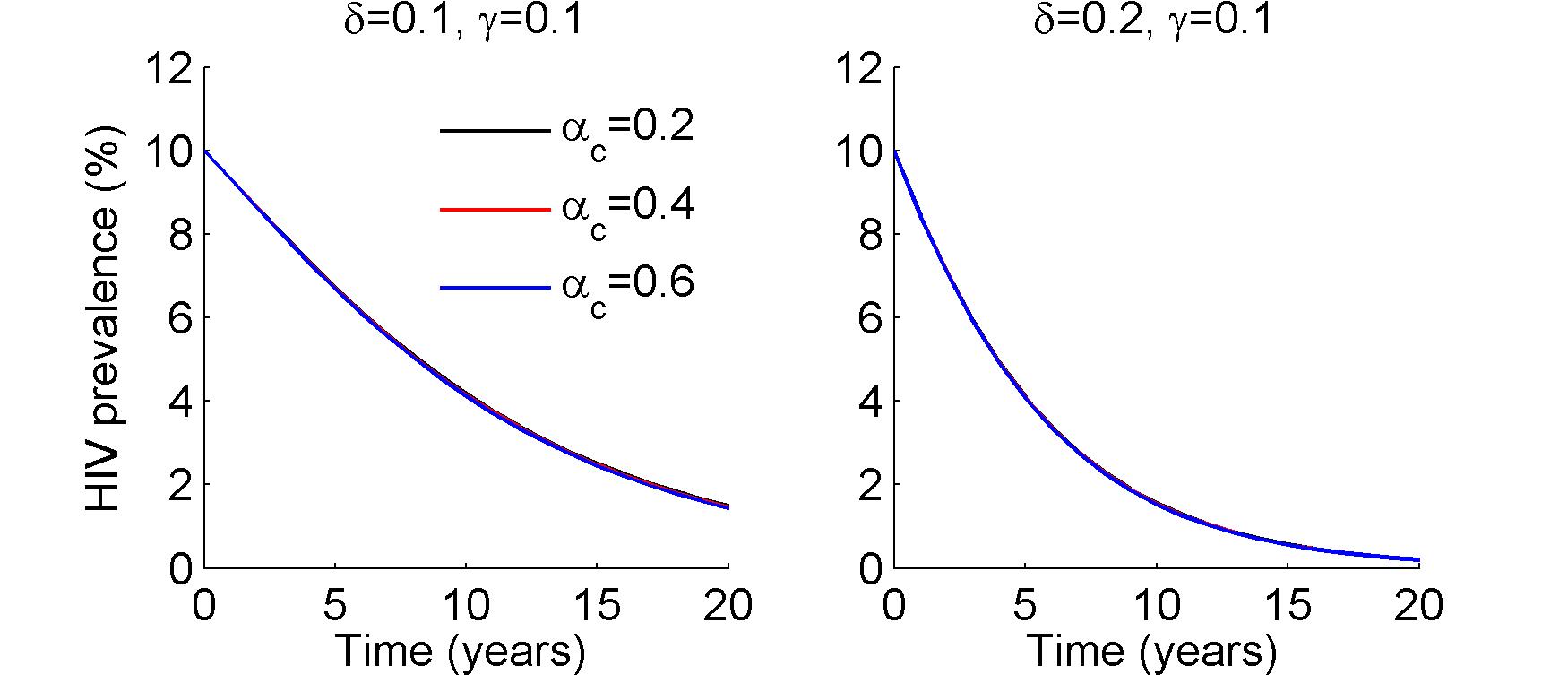


**Cure is contingent upon ART**

**Cure is not contingent upon ART**

C)

D)

**Figure S6. Scenarios assuming that cured individuals are partially protected against reinfection with HIV.** Dynamics of HIV prevalence over 20 years when A,C) 10% and B,D) 20% of the patients on ART (top) or of all HIV infected (bottom) are cured annually and cured individuals are partially protected against reinfection for 10 years. It is also assumed that enhanced HIV prevention is not available (k=0) and that 10% of the untreated individuals initiate ART annually (τ=0.1).

**Sensitivity Analysis**


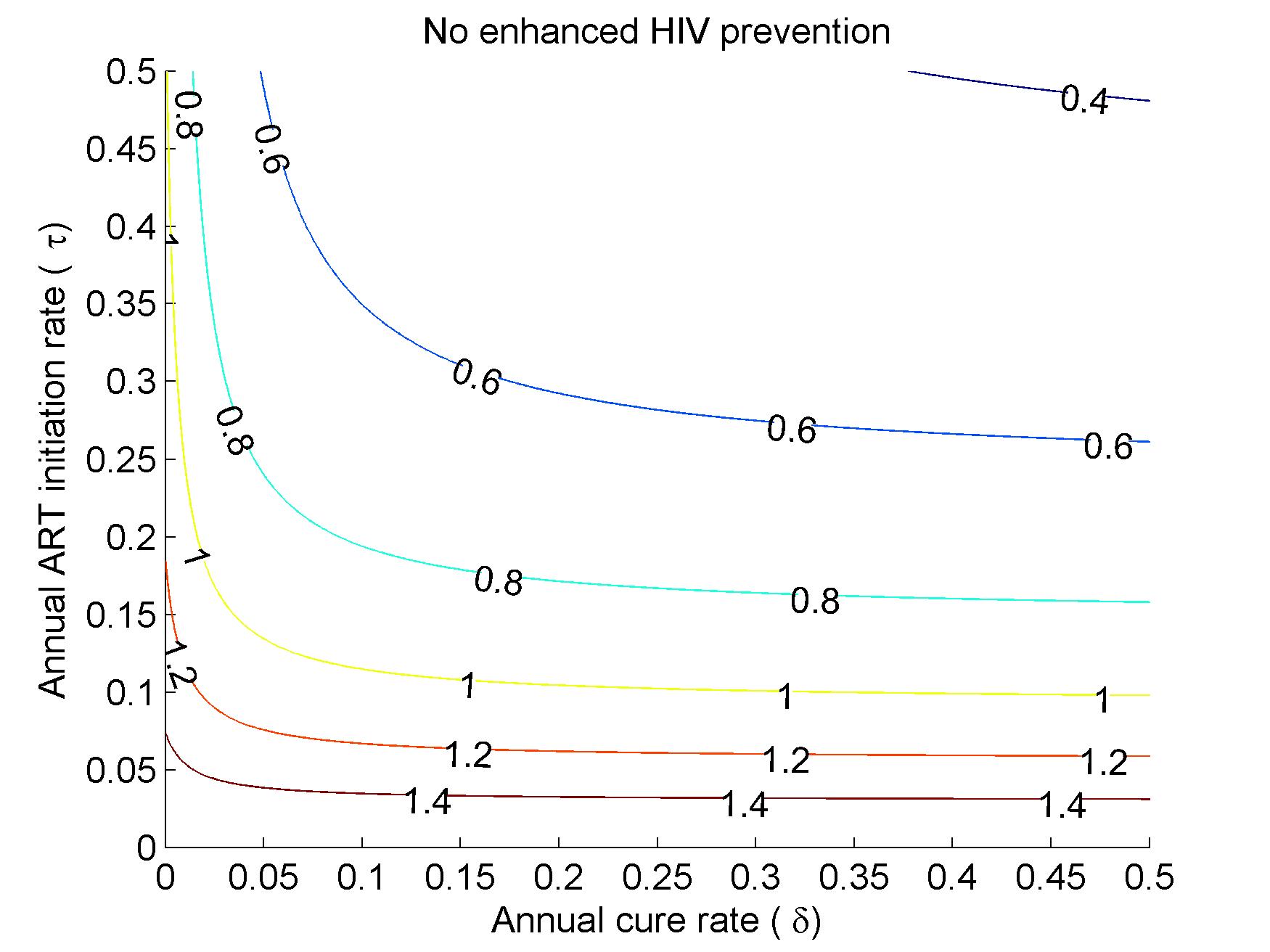

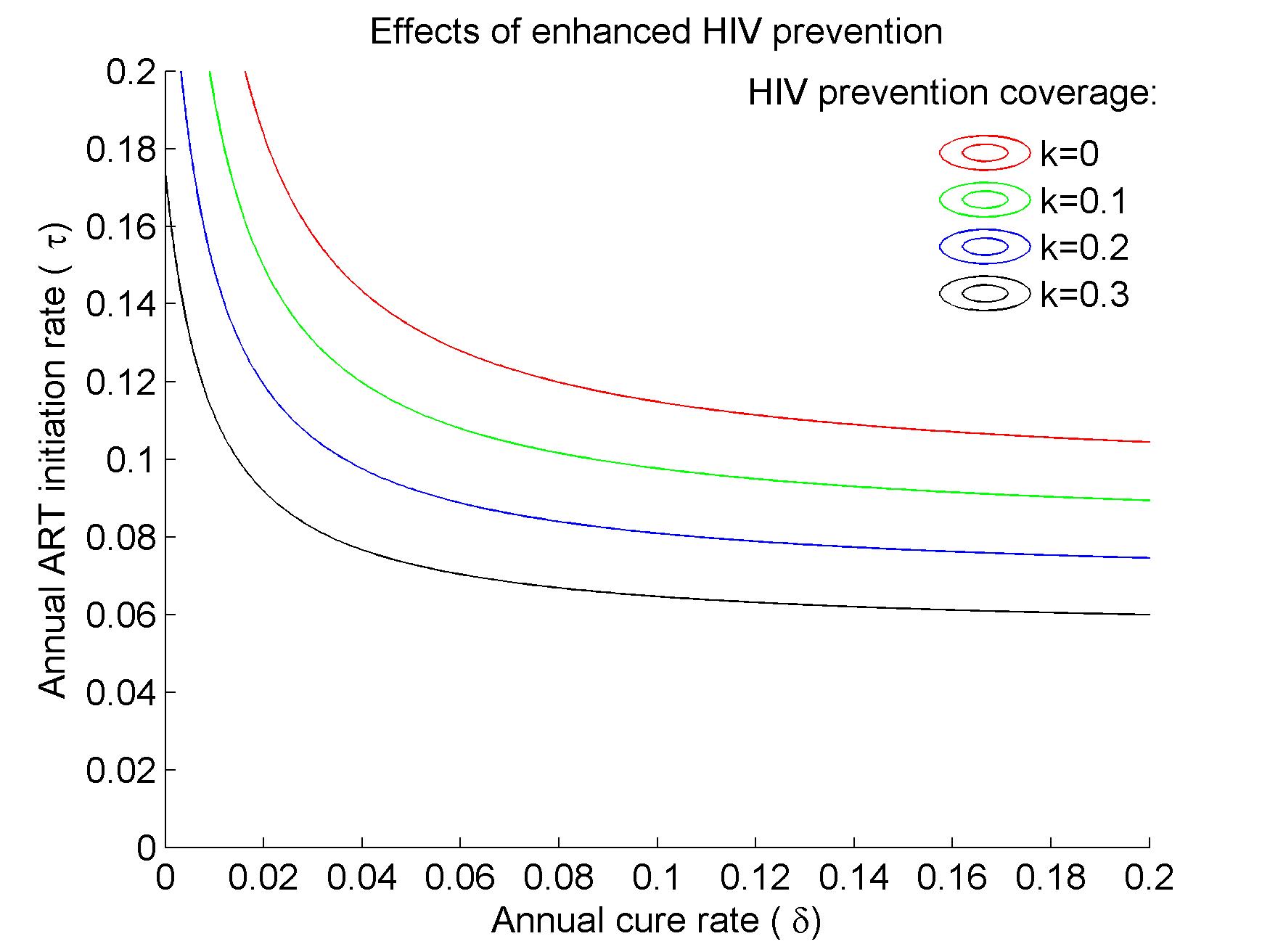


A)

B)


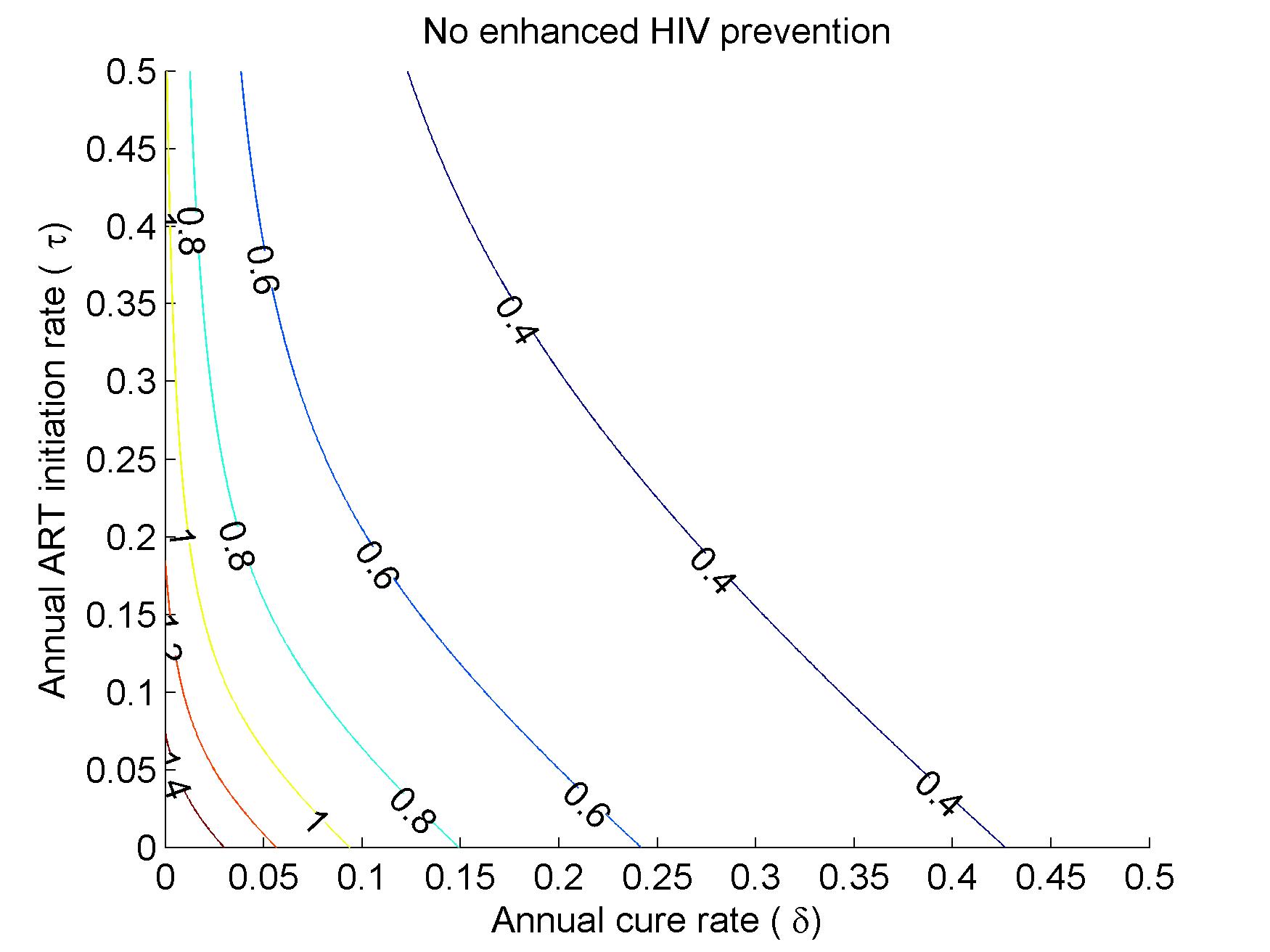

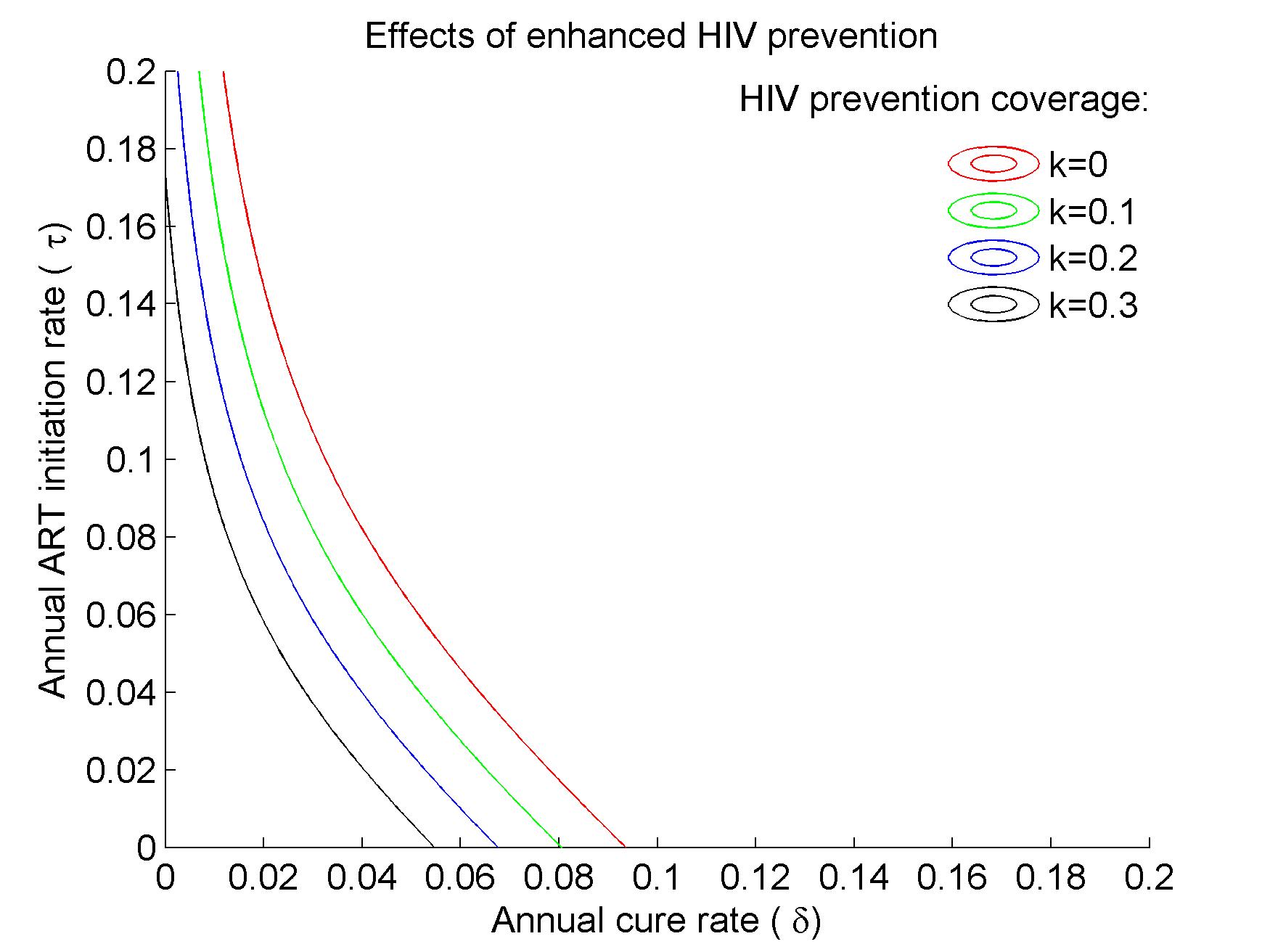


C)

D)

**Cure is contingent upon ART**

**Cure is not contingent upon ART**

**Figure S7. Scenarios assuming 90% effective ART.** A,C) Values of the basic reproduction number (R_0_) assuming different annual treatment and cure rates assuming that PrEP is not available (k=0). B,D) Effects of HIV prevention coverage (k) on HIV eradication (curves represent R_0_=1). **Note that up to 35% annual ART rate are not enough to support HIV elimination in absence of cure. In comparison less than 12% ART initiation rate is needed for HIV eradication if 10% of infected on ART are cured annually while curing 10% of all HIV-infected annually is enough to stop the epidemic even without ART initiation.**


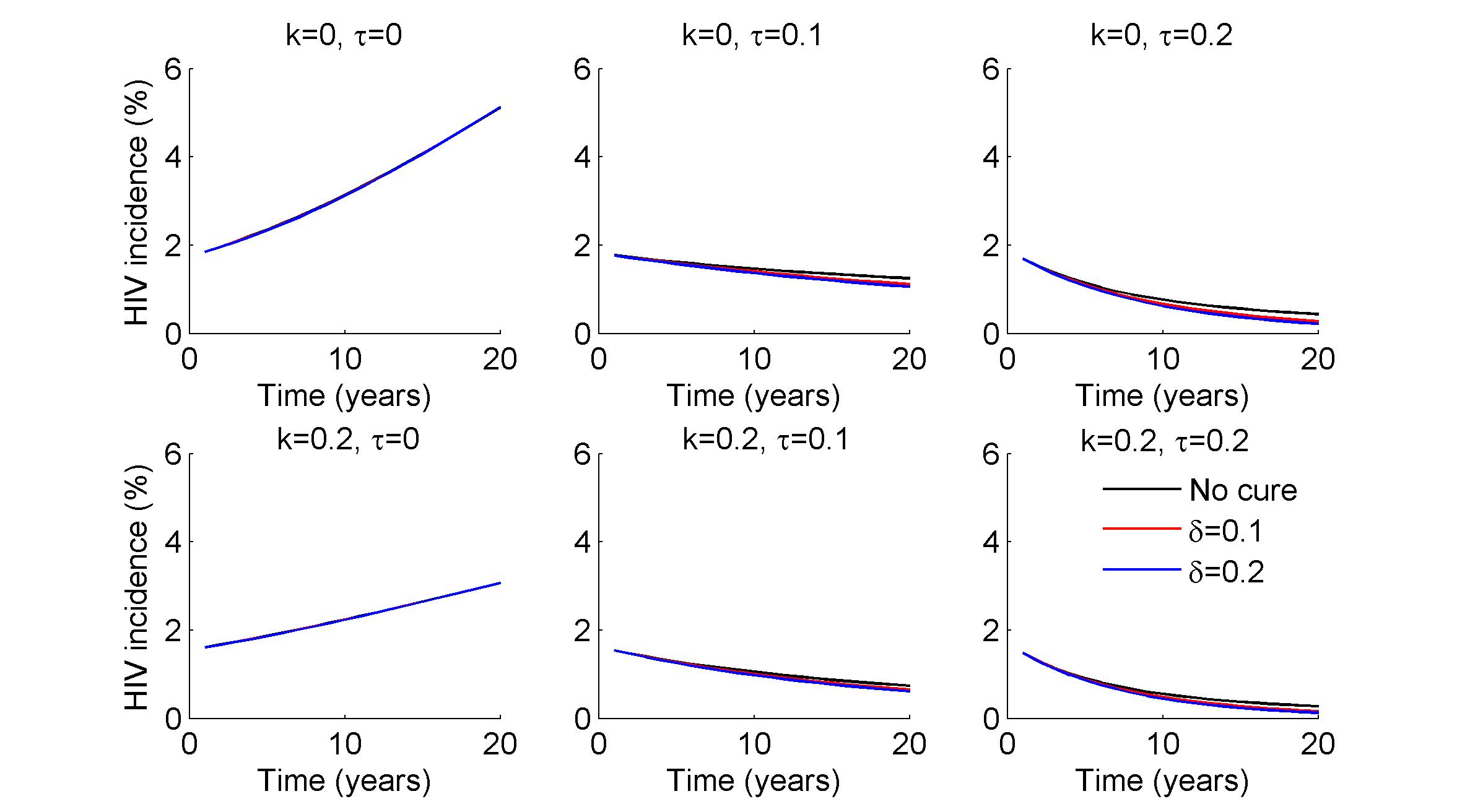


**Figure S8. Scenarios assuming 90% effective ART.** Dynamics of HIV incidence over 20 years for different combinations of HIV prevention coverage (k), ART initiation (τ) and cure (δ) rates.


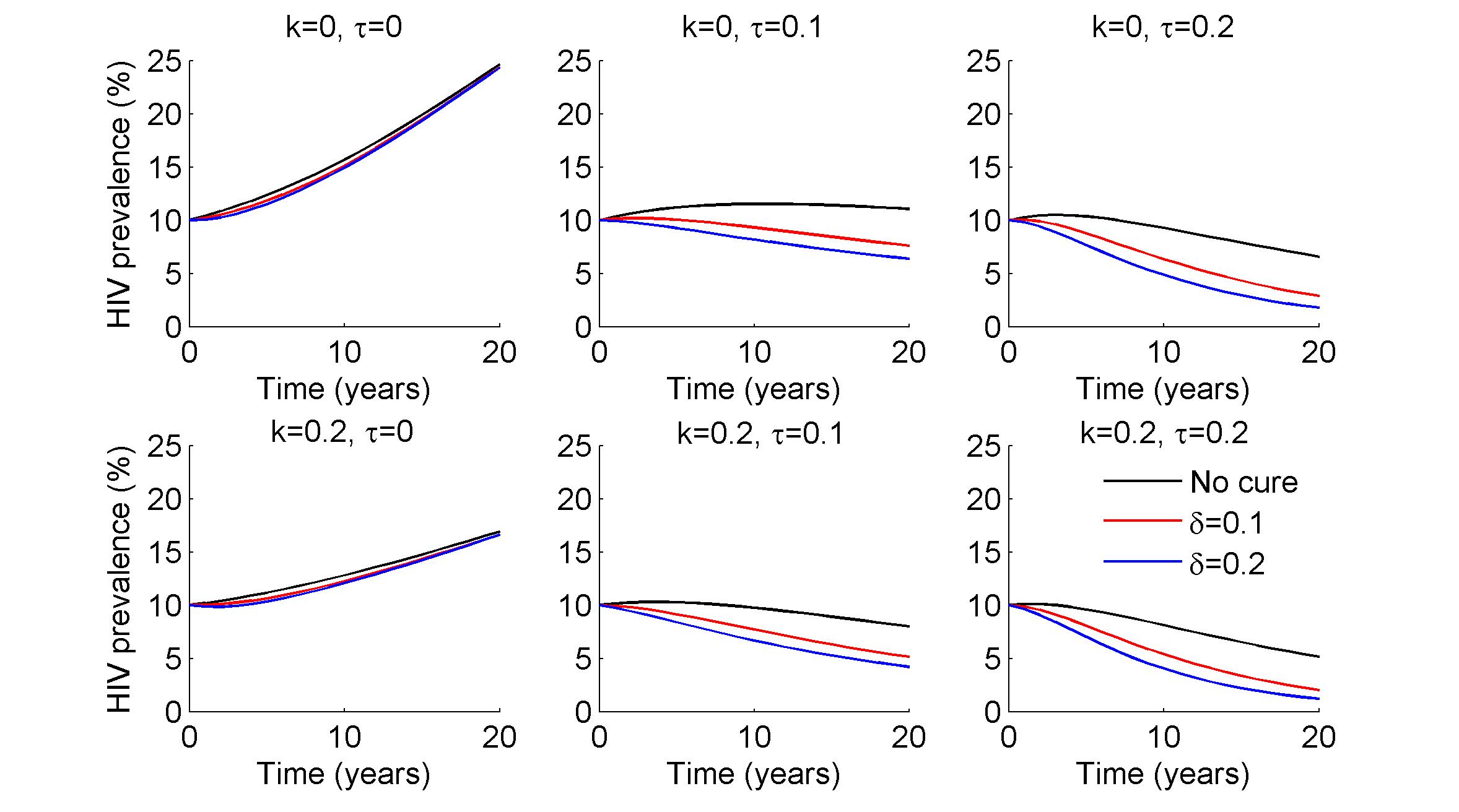


**Figure S9. Scenarios assuming 90% effective ART.** Dynamics of HIV prevalence measured as percentage HIV infected in the population over 20 years for different combinations of HIV prevention coverage (k), ART initiation (τ) and cure (δ) rates.


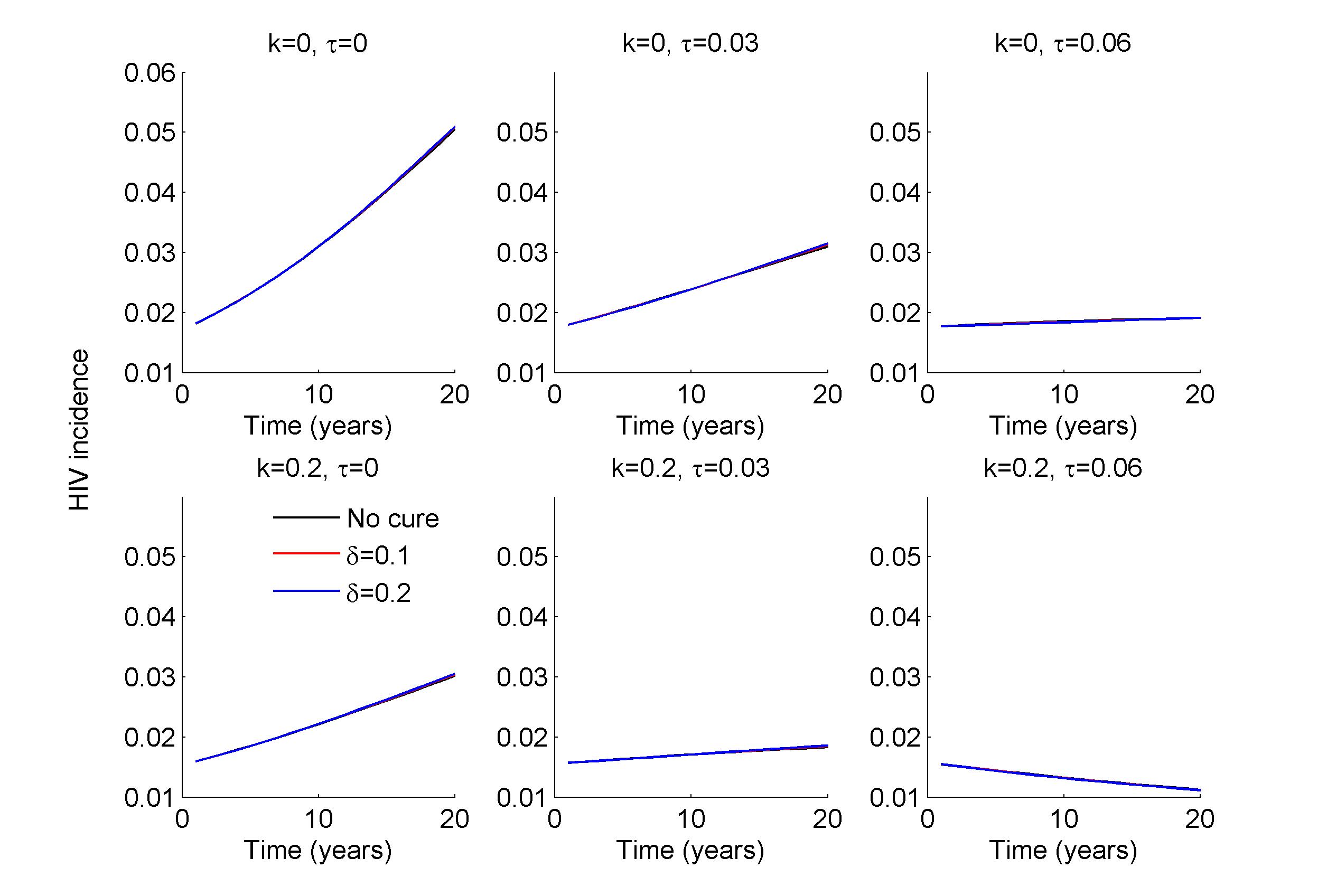


**Figure S10. Scenarios assuming lower ART initiation rates.** Dynamics of HIV incidence over 20 years for different combinations of HIV prevention coverage (k), ART initiation (τ) and cure (δ) rates.

**Figure S11. Scenarios assuming lower ART initiation treatment rates.** Dynamics of HIV prevalence measured as percentage HIV infected in the population over 20 years for different combinations of HIV prevention coverage (k), ART initiation (τ) and cure (δ) rates.


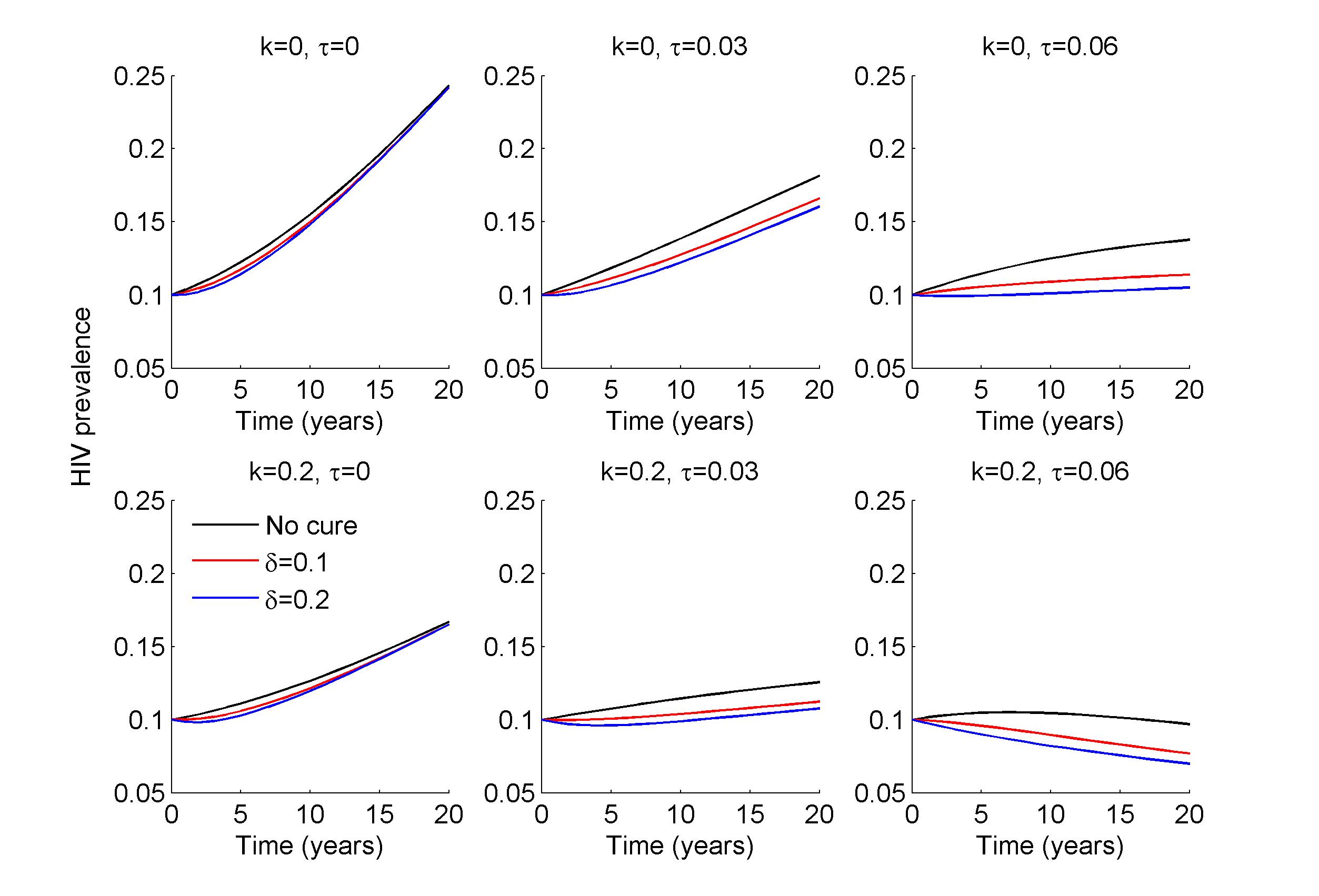

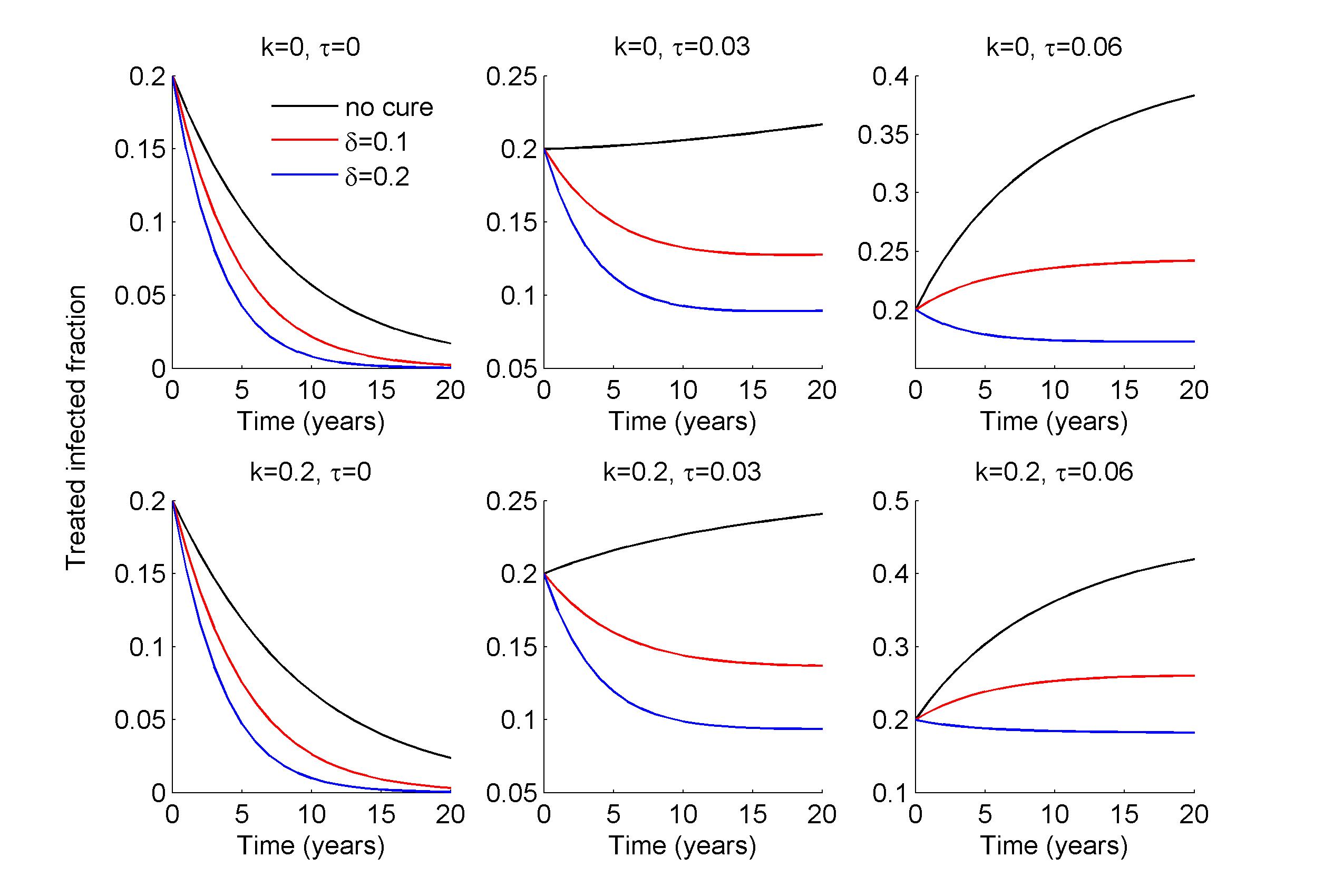


**Figure S12. Scenarios assuming lower ART initiation rates.** Dynamics of the fraction of infected individual on ART over 20 years for different combinations of HIV prevention coverage (k), ART initiation (τ) and cure (δ) rates.

References

1. Boily, M.C., et al., *Heterosexual risk of HIV-1 infection per sexual act: systematic review and meta-analysis of observational studies.* Lancet Infectious Diseases, 2009. **9**(2): p. 118-129.

2. Ferry, B., et al., *Comparison of key parameters of sexual behaviour in four African urban populations with different levels of HIV infection.* AIDS, 2001. **15**: p. S41-S50.

3. Statistics South Africa, *Mortality and causes of death in South Africa, 2011: Findings from death notification*. 2014: Stats SA Library Cataloguing-in-Publication (CIP) Data.

4. Morgan, D., et al., *HIV-1 infection in rural Africa: is there a difference in median time to AIDS and survival compared with that in industrialized countries?* AIDS, 2002. **16**(4): p. 597-603.

5. Porter, K. and B. Zaba, *The empirical evidence for the impact of HIV on adult mortality in the developing world: data from serological studies.* AIDS, 2004. **18**: p. S9-S17.

6. Alemu, A.W. and M.S. Sebastian, *Determinants of survival in adult HIV patients on antiretroviral therapy in Oromiyaa, Ethiopia.* Global Health Action, 2010. **3**: p. 10.3402/gha.v3i0.5398-.

7. Bhatta, L., et al., *Survival on antiretroviral treatment among adult HIV-infected patients in Nepal: a retrospective cohort study in far-western Region, 2006-2011.* BMC Infectious Diseases, 2013. **13**(1): p. 604.

8. Johnson, L., et al., *Sexual behaviour patterns in South Africa and their association with the spread of HIV: insights from a mathematical model.* Demographic Research, 2009. **21**(11): p. 289-340.

9. Kalichman, S.C., et al., *Heterosexual anal intercourse among community and clinical settings in Cape Town, South Africa.* Sexually Transmitted Infections, 2009. **85**(6): p. 411-415.

10. Todd, J., et al., *Reported number of sexual partners: comparison of data from four African longitudinal studies.* Sexually Transmitted Infections, 2009. **85**(Suppl 1): p. i72-i80.

11. Nesri Padayatch, K.N., Halima Dawood, Ayesha BM Kharsany, Quarraisha Abdool Karim, *A Review of Progress on HIV, AIDS and Tuberculosis*, in *South African Health Review 2010*, F.S. Padarath A, Editor. 2010, Health Systems Trust: Durban, South Africa.

12. Statistics South Africa *Mid-year population estimates*. 2013.

13. Grant, R.M., et al., *Preexposure Chemoprophylaxis for HIV Prevention in Men Who Have Sex with Men.* New England Journal of Medicine, 2010. **363**(27): p. 2587-2599.

14. Baeten, J.M., et al., *Antiretroviral Prophylaxis for HIV Prevention in Heterosexual Men and Women.* New England Journal of Medicine, 2012. **367**(5): p. 399-410.

15. Cohen, M.S., et al., *Prevention of HIV-1 Infection with Early Antiretroviral Therapy.* New England Journal Of Medicine, 2011. **365**(6): p. 493-505.
